# Ageing as a software design flaw

**DOI:** 10.1186/s13059-023-02888-y

**Published:** 2023-03-28

**Authors:** João Pedro de Magalhães

**Affiliations:** Genomics of Ageing and Rejuvenation Lab, Institute of Inflammation and Ageing, University of Birmingham, Birmingham, B15 2WB UK

**Keywords:** Antagonistic pleiotropy, Genome, Information theory, Longevity, Programmed ageing

## Abstract

Ageing is inherent to all human beings, yet why we age remains a hotly contested topic. Most mechanistic explanations of ageing posit that ageing is caused by the accumulation of one or more forms of molecular damage. Here, I propose that we age not because of inevitable damage to the hardware but rather because of intrinsic design flaws in the software, defined as the DNA code that orchestrates how a single cell develops into an adult organism. As the developmental software runs, its sequence of events is reflected in shifting cellular epigenetic states. Overall, I suggest that to understand ageing we need to decode our software and the flow of epigenetic information throughout the life course.

**Supplementary Information:**

The online version contains supplementary material available at 10.1186/s13059-023-02888-y.

## Introduction

Ageing can be defined as an inevitable and progressive deterioration of physiological function, accompanied by an increase in vulnerability and mortality with age [1]. The human ageing process entails countless changes at multiple biological levels, degenerative changes in virtually all organs and body systems, and increased susceptibility to several diseases, such as cardiovascular diseases, cancer, neurodegenerative diseases, type II diabetes, and many infectious diseases [2]. Although ageing is integral to human biology and has a major impact on society and medicine, it remains at the mechanistic level a poorly understood process.

Many theories of why we age have been proposed, including damaged-based and programmatic theories, with the former currently more widely accepted and studied [1, 3–6]. Most damage-based theories postulate that inefficient repair mechanisms result in singular or multiple, and often interacting, forms of damage accumulation. Although damage can be broadly defined as any change that affects function, here I refer more specifically to molecular damage hypothesized to drive ageing, such as by-products of metabolism, unwanted chemical modifications, and other types of molecular damage affecting crucial cellular components like the genome, telomeres, mitochondria, and proteins [5, 7, 8]. By contrast, programmatic theories argue that ageing results from predetermined mechanisms encoded in the genome, rather than stochastic damage accumulation [9–14]. There has also been considerable progress in manipulating ageing in model organisms using genetic, dietary, and pharmacological interventions [2, 15–17]. Despite these advances, why human beings age remains a mystery subject to intense debate [18].

In recent years, the development of epigenetic clocks has shown that a relatively small number of methylation sites, some becoming hypermethylated and others becoming hypomethylated with age, can predict human chronological age with surprisingly high accuracy [19–21]. Epigenetic clocks can also predict mortality risk in humans, and universal mammalian epigenetic clocks can predict the age of individuals from mammalian species with vastly different lifespans [21–25]. Epigenetic clocks tick throughout the entire human lifespan, starting at conception, and they tick in normal human cells in vitro, but not in embryonic or pluripotent cells [19, 26]. Reprogramming with Yamanaka factors resets epigenetic clocks to zero [19, 27]. Taken together, these findings are surprising because of the high accuracy of epigenetic clocks and their association with mortality as well as the clock’s reset with reprogramming. The mechanisms underlying epigenetic clocks are contentious, however, and whether they are drivers or passengers of ageing is unknown.

The concept of information in biology has a long history [28], and biological systems can be seen as highly complex information systems. Likewise, the idea that ageing could be linked to information decay or loss has been proposed, in particular in the context of the information theory of ageing [29–31]. According to this theory, loss of genetic [29] or epigenetic [30, 31] information with age, driven by DNA damage, is the primary cause of ageing. One hypothesis is that errors accrue in the DNA, corrupting the information in the genome and ultimately disrupting tissue homeostasis and causing ageing [32, 33]. More broadly, the idea that errors or damage to one or more biological types of hardware, including the DNA, accumulate and drive the process of ageing has been prevalent for decades. By hardware I encompass all elements of biological systems, including organs, tissues and the basic unit of life, the cell, and its structures (mitochondria, telomeres, proteins, DNA, and so on), most of which have at some point been hypothesized to be important in ageing [5, 7].

What if, however, the processes that cause ageing are not a product of inevitable molecular damage but rather intrinsic features of the software? In this context, I define software as the genetic program, the DNA code that orchestrates how a single cell becomes an adult human being capable of reproducing, ultimately our evolutionary purpose. Herein, I present and explore the hypothesis that perhaps ageing is not a result of inevitable wear and tear or accumulated molecular damage in the hardware but rather that ageing is caused by design flaws in the software itself. I discuss manipulations of ageing and how they support this hypothesis, acknowledge exceptions, and lastly, propose areas of future study.

## From the digital code to ageing

Clearly, there is a software program, encoded in the DNA, that is far more advanced, with much greater algorithmic complexity, than any computer program. Another big difference is that the genetic software program builds its own hardware. Indeed, the human genome encodes many biological instructions and features, from the basic biochemistry of life to the wiring of the human brain and how the immune system fights pathogens. In the context of this essay, I focus on one aspect of the software, and that is the program that sets in motion the extraordinary changes that occur from conception until adulthood. As such, this developmental software program is the sequence of instructions for producing a reproductively competent adult. John Maynard Smith, in fact, argued that the genome could be seen as a developmental program, and “genes carrying information during development” [28]. It remains poorly understood how exactly a single cell (egg) develops into an embryo and then turns into a foetus which later becomes a newborn that in turn will grow and develop into an adult organism made up of billions of cells with many different identities and functions. Nonetheless, an enormous combination of changes at different paces must occur in the many cell types and organs in the body throughout the process of development. What if it is not repair and protective mechanisms that define the pace of ageing but rather the information that regulates development and becomes destructive later in life [9]. In other words, some genetically regulated processes set in motion during development become detrimental in adulthood and cause degeneration and loss of function. Examples include changes in cell composition and physiological signals (e.g. hormonal changes) or the continual growth of particular tissues, as observed in presbyopia that is thought to result from the continuous growth of eye lenses [34, 35]. My hypothesis is that intrinsic flaws in the developmental software program are the major driver of what we call ageing.

The accuracy in humans and across mammalian species of epigenetic clocks, even if their mechanistic basis is not well understood, point towards conserved, fundamental processes at play during development and ageing. Of note, the Horvath clock based on 353 CpG methylation sites correlates with human chronological age in multiple tissues; it is accurate in children and adults and correlates with gestational age [19]. Strikingly, processes associated with genes in the vicinity of epigenetic clock methylation sites are often related to growth and development [21, 22, 36]. For example, target sites of polycomb repressive complex 2 (*PRC2*), which plays a major role in development, are enriched in epigenetic clocks [21, 22, 37]. Earlier studies of DNA methylation changes in ageing also found a significant number of developmental genes and processes [38–40]. Moreover, ageing is generally characterized by genome-wide hypomethylation and promoter-specific hypermethylation [41], which suggests programmatic (rather than random) processes. To be clear, epigenetic clocks likely reflect ageing mechanisms but also ageing-independent processes. Likewise, we have a limited understanding of the biological basis of epigenetic clocks, of which mechanisms drive those clocks or of how many cells and which cell type(s) contribute to the clocks. Be that as it may, the fact that the clocks are ticking from very soon after conception and that they are so accurate from such early developmental stages until old age point towards a link between developmental programs and ageing. To quote Raj and Horvath [42]: “while the speed of ageing can, and is affected by external factors, the essence of the ageing process itself is an integral part of, and the consequence of the development of life.”

**Fig. 1:**
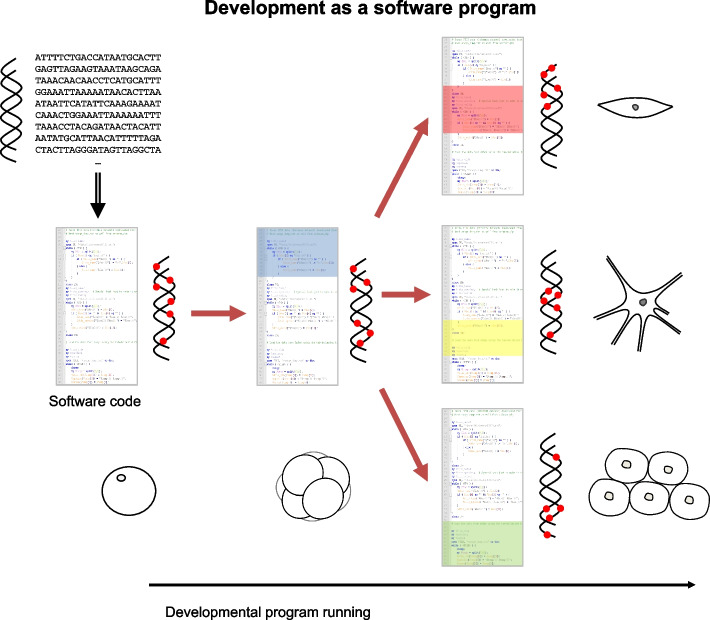
Ontogeny as a software program encoded in the genome and running in the epigenome. The developmental software program is encoded in the DNA sequence. As the program runs, numerous subroutines are called and operate in different spatial and temporal contexts. The epigenome, at the level of DNA methylation (shown), histone modifications, chromatin structure and noncoding RNAs (not shown), acts in cells as the software’s data area. Differences in space and time in the running of the developmental software program (represented by different colours in the code) are also embedded in the epigenome/data area that is read and written by the software and dictates when and which subroutines are run—e.g. different gene expression programs, transcription factors, signalling pathways and protein levels (not shown)—and ultimately determines cellular phenotypes

While the developmental software program is encoded in the DNA, its sequential running is not linked to changes in the DNA sequence but rather to epigenetic changes that activate or shut down gene expression programs (subroutines in the software) that in turn result in cellular functions and phenotypes. In other words, the software code is in the DNA sequence but runs in the epigenome, which can be seen as a data area (Fig. 1). As such, I speculate that some epigenetic clocks like the Horvath clock partly reflect the running of the developmental software program. To put it another way, the epigenome encodes the passage of time in cells during development and, I argue, during ageing. In this regard, methylation clocks are likely the tip of the iceberg concerning epigenetic changes that modulate development and ageing. For one, despite recent advances in omics technologies, what we can quantify in biological systems is still limited. Furthermore, epigenetic clocks are composed of only a small fraction of methylation changes with age, in turn only a fraction of epigenetic changes during ageing that have been characterized [43–45]. As developmental programs run, several layers of epigenetic regulation control cell function, differentiation, and behaviour in turn affecting signalling pathways, tissues, and organs. As such, it seems likely that multiple types of epigenetic changes (including methylation, histone modifications, chromatin structure and noncoding RNAs) store information of the running of the developmental software program during the life course.

Another reason to consider that human ageing may be an outcome of our developmental software program is the observation that most ageing phenotypes are not stochastic or random, but gradual and predictable. There are exceptions, like tumours (see below) and cerebral cavernous malformations [46], but most ageing changes like loss of muscle mass (sarcopenia), decreased wound healing, grey hair, bone thinning, arterial stiffness and vascular ageing, thymus involution, and loss of function in most organs are gradual, widespread, and—although they can often be delayed by lifestyle and environmental factors—inevitable. Ageing changes also typically do not vary significantly within individuals. For example, in men, greying beard hairs tends to be symmetrical [47]. Likewise, as above-mentioned, epigenetic clocks are surprisingly accurate throughout the entire lifespan, which again does not fit the idea that ageing is a product of entropy breaking down the body. Quite the opposite, the strong, deterministic patterns we see in ageing suggest an underlying driver.

**Fig. 2:**
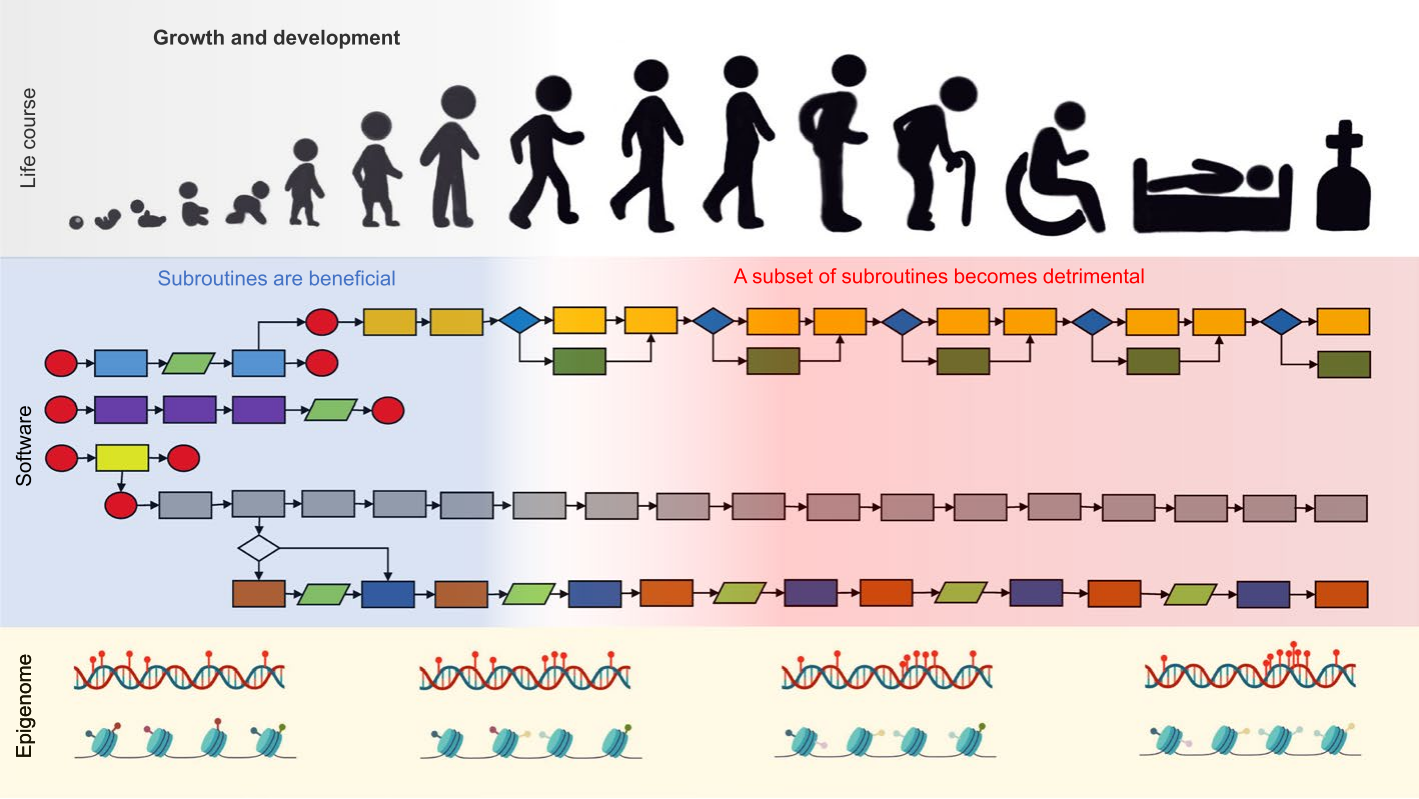
Ageing as the result of software design flaws. The developmental software program is a set of instructions or interconnected subroutines with numerous inputs and outputs that trigger a complicated cascade of events that drive growth and development. Because this software program is optimized for reproduction, however, it fails to deactivate a subset of its subroutines which are beneficial during development (blue shading) but then become detrimental later in life (red shading). With age, such subroutines could gradually lead to the inappropriate activation or inactivation of genes, pathways, and processes that drive ageing phenotypes. The running of the developmental software program is reflected in the epigenome, the software’s data area. Human life course drawing by Alice C Magalhaes. Epigenome figure created with BioRender.com

My proposal is therefore that the ageing process and phenotype (with some exceptions discussed below) is not driven by passive random molecular damage but rather by the developmental software program optimized for reproduction that becomes detrimental later in life, as a form of antagonistic pleiotropy [48]. For the avoidance of doubt, the software becomes detrimental later in life not because it was moulded directly by natural selection for such a purpose but rather because after reproduction the force of natural selection declines with age and the software design flaws become apparent [9]. Ageing is a program that is set in motion shortly after conception, though not a program that was intentionally designed to harm us [10]. Because ontogeny and how the developmental software run are intricately linked to epigenetic processes that define cell function, differentiation and identity, epigenetic changes during development continue in adulthood and reflect the running of the developmental program. That is to say, gradual changes in epigenetic states set in motion during development continue in adulthood, as observed in epigenetic clocks [19, 21], as well as other studies [10, 39, 49]. For example, one study in mouse tissues found that DNA methylation changes in adulthood appear to be an extension of changes during growth, rather than changes driven by deterioration that start after adulthood [40]. We age in part because of intrinsic design flaws in the software running in the epigenome (Fig. 2).

**Fig. 3:**
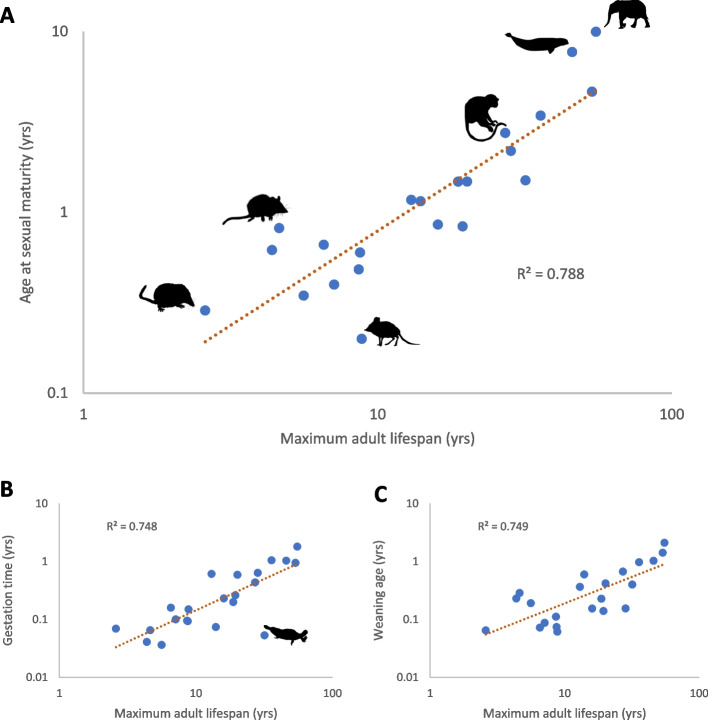
Strong correlation between longevity and developmental traits in mammalian families. **A** Correlation between maximum adult lifespan and age at sexual maturity (*r*^*2*^ = 0.788). **B** Correlation between maximum adult lifespan and gestation time (*r*^*2*^ = 0.748); monotremes, the only egg-laying clade of mammals, are an outlier. **C** Correlation between maximum adult lifespan and weaning age (*r*^*2*^ = 0.749). Only families with at least three species are shown (*n* = 22). Maximum adult lifespan = maximum lifespan minus age at sexual maturity. Data from AnAge build 14 [15]. Silhouettes from phylopic.org

If ageing is a run-on from developmental programs, an unintended consequence of the software running via the epigenome on every cell of every individual, this would explain the wide variety of species differences in ageing amongst mammals. It has long been a mystery why closely related species can age much faster than others even in optimal environmental conditions, like mice that age 20–30 faster than human beings despite a similar basic biochemistry and biology [35, 50]. Even amongst primates there are marked differences in lifespan, for example rhesus monkeys are considered old by age 30, and marmosets when they are 8 years old [51]. Interestingly, between mammalian species there is a very strong correlation between age at sexual maturity and the remaining lifespan, irrespective of metabolic rate or body size [52]. In other words, how long it takes animals of a species, on average, to reach reproductive age is highly predictive of how long they live afterwards before they age and die (Fig. 3); strong correlations are observed between adult lifespan and not only age at sexual maturity (Fig. 3A) but also gestation time (Fig. 3B) and weaning age (Fig. 3C). As such, what if the chain of events that occurs between adulthood and old age is set in motion because of design flaws in the developmental software program? Given the very strong correlation across species between age at maturity and the resulting lifespan (Fig. 3), if the pace of ageing is mechanistically linked to the pace of development, then this would explain species differences in ageing. Simply put, I hypothesize that a mouse develops and ages 20-30 times faster than a human being because its developmental software program runs 20-30 faster than in a human being.

## Cancer as an exception that results in a cellular balancing act

Ageing entails a vast array of changes and pathologies. It would be naïve to assume that software design flaws are responsible for all aspects of the ageing phenotype. Indeed, there is one major age-related disease that is likely due to stochastic damage: cancer. In contrast to most other ageing phenotypes that, as abovementioned, are gradual and to a certain extent predictable, cancer is heterogeneous and erratic. It is largely accepted that, even though multiple processes (including ageing processes) can play a role in tumourigenesis [53], cancer is primarily driven by damage, specifically random DNA damage and mutations. Recent studies, in fact, show that older people may have billions of cells with oncogenic mutations [54]. Cancer is also widespread amongst metazoans and mammals [55]. At a fundamental level, complex multicellular organisms are susceptible to rogue cells that proliferate uncontrollably and avoiding and stopping such rogue cells is essential for survival.

Because cancer is such an inevitable and basic threat to animals, and particularly mammals with their multiple dividing cell types, there is very strong selection to prevent cancer in young animals [56]. This is reflected in numerous adaptions in long-lived mammals, such as shorter telomeres and repressed telomerase [57] as well as a slower mutation rate accumulation with age [58]. But cancer prevention must also be reflected in the developmental software in the form of early life processes that minimize cancer risk. As the number of cells in an organism increases rapidly during development, the probability of individual cells becoming tumours must be reduced. Therefore, as proposed by others [59, 60], one major axis of developmental processes that become detrimental later in life likely reflects changes in cells during early development aimed at tumour suppression that continue in adulthood. This might include, I speculate, changes in the proportion, number, or activity of stem cells, in the proliferative capacity of cells, in cellular plasticity and/or in cell differentiation. Indeed, we know of one example in the form of telomerase expression that is high during early stages of development and lower in most adult tissues [61], presumably for tumour suppression. The developmental transitions and changes designed to prevent cancer may result in the loss of regenerative potential or repair capacity later in life, leading or at least contributing to degenerative diseases [59, 60]. In other words, the developmental software program must prevent cancer during ontogeny by reducing the number of cancer-prone cells or restraining cell functions associated with tumourigenesis, and its run-on becomes detrimental during ageing. Indeed, it has been suggested that perhaps epigenetic clocks reflect some measure of stem cell differentiation [21].

Some simpler organisms, like Hydra, strikingly do not inhibit developmental plasticity throughout their development allowing them to regenerate as well as reproduce asexually through budding [62]. Even though Hydra suffer from molecular damage like mutations [63] and can develop cancer [64], these animals appear not to age. The medusa *Turritopsis nutricula*, also called the immortal jellyfish, is an even more extreme case of developmental plasticity in that mature individuals can reverse their life cycle and become juveniles [65]. Interestingly, many species, like some salamanders [66], can regenerate limbs via reactivation of developmental pathways; in fact, it appears as if development is more plastic in other taxa, including reptiles and amphibians, when compared to mammals [9]; and as a side note, why cancer is not more prevalent in such taxa is unknown. Mammals, perhaps by chance or because of their evolutionary history [67], have in general a less plastic development and must establish a balance between development, repair, and cancer, resulting in a loss of regenerative potential following development [60]. Put differently, stem cells and/or cellular plasticity need to be curbed to avoid cancer in mammals, which results in a loss of replicative and regenerative potential during development that continues in adulthood and contributes to ageing [59]. Indeed, wound healing declines from early development until adulthood and then during adulthood and later in life [68]. By contrast, reprogramming cells with Yamanaka factors back into early developmental stages will render them more cancer prone [69].

Molecular damage to human cells is not inevitable. We originate in a single fertilized egg that undergoes massive cell division and proliferation during early development. Some human cell types, like telomerase-immortalized fibroblasts, can also proliferate in culture indefinitely [70]. It is possible that as the developmental software program runs, it causes a downregulation of repair mechanisms later in life, like DNA repair, allowing for the accumulation of some forms of molecular damage. The abovementioned downregulation of telomerase and telomere repair during development could be seen as one such cases. Therefore, although cancer is a product of random entropic processes and does not appear to be driven by programmatic mechanisms that cause most other facets of ageing, the running of the developmental software may have some impact on cancer, the degree of which remains to be established.

## Manipulations of ageing change the software’s runtime

Genetic, dietary, and pharmacological manipulations of ageing and longevity in model organisms are arguably one of the major breakthroughs in geroscience. Over 2000 genes have been reported to modulate longevity in model organisms [15], and the observation that single gene manipulations can retard the whole ageing process supports the idea that ageing is a coordinated process [71]. Genes retarding or accelerating ageing can be grouped into common pathways, such as growth hormone/insulin-like growth factor 1 (GH/IGF1), cell cycle regulation, and mTOR signalling [72]. There are also over 1000 drugs or compounds reported to extend lifespan in model organisms [15]. These drugs target a variety of processes that, by and large, reflect those thought to be associated with ageing, like cellular senescence, mitochondrial dysfunction, oxidative stress, and inflammation [16]. But how do these ageing manipulations fit the ageing as a software design flaw hypothesis?

First, it is important to note that, because many different factors contribute to mortality, longevity manipulations do not necessarily impact the ageing process [50, 73–75]. For example, in mice that mostly die of cancer, an intervention that reduces cancer mortality will extend lifespan, without necessarily having an impact on function or on any other aspect of the ageing phenotype [74]. Longevity thus reflects different processes, one of which is ageing. In addition, longevity manipulations may have strain-specific effects that obscure an impact on organismal ageing [76]. A detailed discussion of longevity manipulations in model organisms and their impact (or not) on ageing is outside of the scope of this article. Nonetheless, it is important to reiterate that not all longevity manipulations, whether they are genetic, dietary, or pharmacological, modulate the ageing process, and hence care is needed when interpreting results from longevity interventions.

**Fig. 4:**
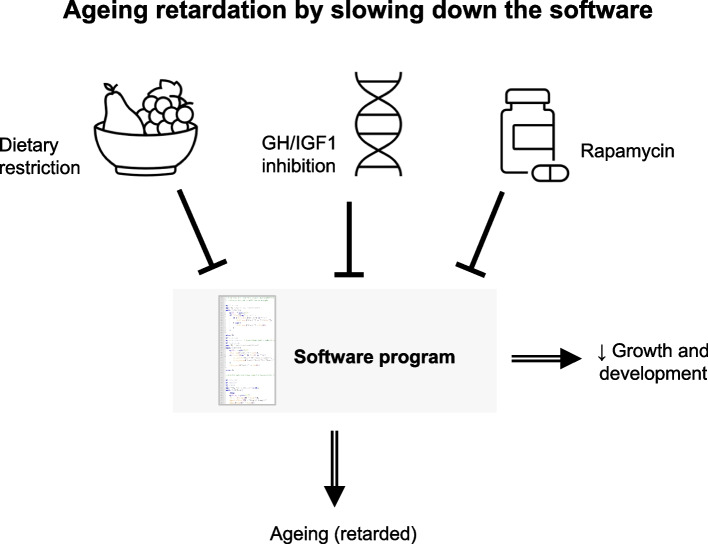
The most well-established longevity manipulations in mice may retard ageing by slowing down the developmental software program. Dietary restriction, GH/IGF1 inhibition, and rapamycin are, respectively, the major dietary, genetic, and pharmacological life-extending interventions. All these manipulations also regulate growth and development and hence may slow down the running of the developmental software program which in turn retards ageing, supporting the idea of ageing as a software design flaw

With the abovementioned caveats in mind, clearly some pathways and interventions have emerged as major modulators of ageing across model systems (Fig. 4). In particular, the GH/IGF1 pathway is the best characterized pathway that, when inhibited, modulates ageing in animal models, including in rodents [17]. Interestingly, GH/IGF1 is also a major modulator of growth and development, in turn supporting the idea that slowing down the whole developmental program, i.e. slowing down the rate that the developmental software program runs at, slows down ageing [77]. Likewise, dietary restriction is the most widely studied and robust dietary manipulation of ageing, extending lifespan in a variety (but not all) animal models [2, 76, 78]. Just like reduced GH/IGF1 signalling, dietary restriction also slows down growth and development [77]. Therefore, it is striking that the two major known interventions that retard ageing in mammals also retard the pace of development and growth—that is, they slow down the developmental software program (Fig. 4).

Strikingly, the most robust life-extending pharmacological intervention in mammals is rapamycin [16], which also slows down growth. Indeed, rapamycin targets mTOR, a major regulator of cellular metabolism and growth [79], whose inhibition induces pausing of mouse blastocyst development [80], and has been proposed as a key player in program-like ageing [11, 81]. In human cells, rapamycin treatment retards epigenetic ageing [26]. Recent results in mice and invertebrates also show that rapamycin early in development can suppress growth and extend lifespan later in life [82], which suggests a causal relationship between the pace of development and longevity and again support the idea that ageing is an outcome of developmental processes [83]. It is important to acknowledge that rapamycin treatment and dietary restriction later in life also extend lifespan in mice [78], which is expected if developmental software programs running across the life course drive ageing. Overall, the major known manipulations of ageing may work by retarding the developmental software program (Fig. 4).

The idea that developmental factors can impact on ageing has been debated for over a century [84], including as part of a program [14, 85]. Evidence for the existence of programmatic features in ageing has been observed in simple organisms, like yeast [86], which interestingly have also been suggested to age due to loss of epigenetic, rather than genetic, information [31, 44]. In invertebrates, like flies [84, 87] and molluscs [88], there is abundant evidence of a link between development and ageing. Social insects in which the same genome gives rise to phenotypically different castes (i.e. workers and queens) with vastly different lifespans fit the idea that ageing can be determined by developmental programs [89]. Furthermore, the roundworm *C. elegans* has provided plentiful of empirical evidence [90–92], dating back to decades-old observations that developmental arrest in worms can dramatically extend lifespan [93, 94]. Although rare and exceptional, vertebrate species, like the salmon, that reproduce only once before suffering from a rapid degeneration also support the thesis that degenerative changes and death can originate from the developmental software program optimized for reproduction [9, 14, 92]. The observation that, in many poikilotherms, a lower temperature slows down development and ageing [95] further underscores the thesis that the software regulating development also regulates ageing.

In mammals, it has long been observed that certain tissue and physiological age-related changes may be an extension of mechanisms that control earlier development, such as neural and endocrine mechanisms [96, 97]. There is also a large body of work on developmental programming showing that the foetal and neonatal environments can—presumably via epigenetic mechanisms—have a profound impact on various age-related diseases, such as hypertension, diabetes, and obesity [98]. We also know that developmental genes can be detrimental later in life; for example, double homeobox protein 4 (DUX4) plays a role in early embryonic development and is normally epigenetically silenced afterwards, yet its aberrant expression in muscle causes muscular dystrophy in patients [99]. More recent studies using high throughput approaches have also revealed links between changes during development and ageing [10, 40, 49, 100, 101], as highlighted in epigenetic clocks [21, 22, 42]. Other recent studies, for example of hematopoietic stem cells in mice [102] and the aryl hydrocarbon receptor [103], further support the concept that developmental pathways influence ageing phenotypes later in life. Although these studies are encouraging, many open questions remain. Our understanding of mammalian developmental processes is still very limited. In spite of recent results suggesting metabolic regulation contributes to differences in rates of development between mouse and human embryos [104], the causes of differences in developmental rates between species remain largely a mystery. The complexity of human biology itself is beyond our current comprehension; we still lack a detailed understanding of the workings of the amazing molecular machinery within the cell and of how billions of cells interact and come together to form a functioning organism. Therefore, it remains to be elucidated exactly how the developmental software works, how it orchestrates development and eventually which of its programs and their design flaws drive ageing.

## Resetting the clock is akin to a computer restart

**Fig. 5:**
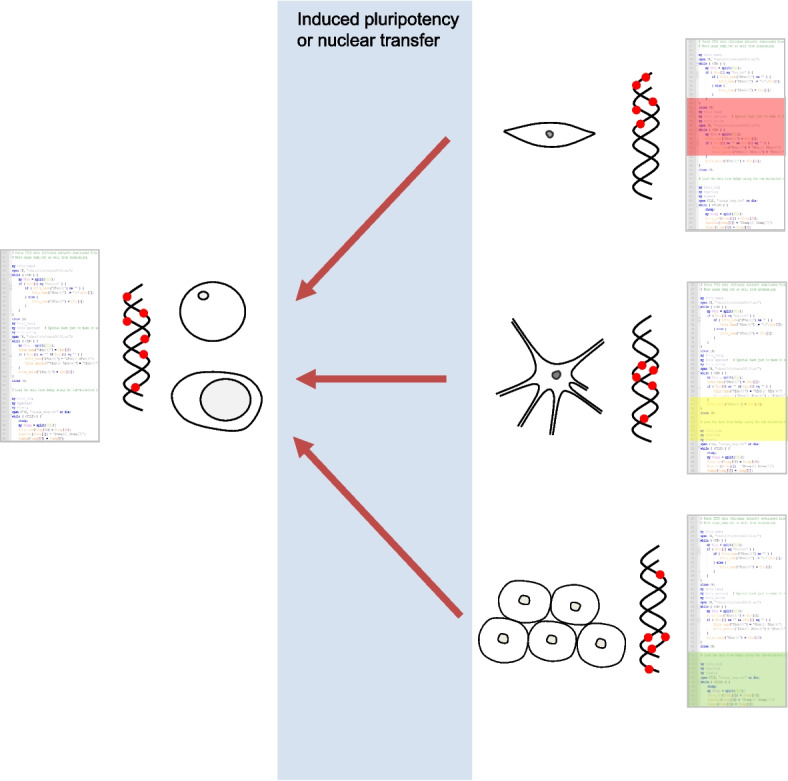
Nuclear transfer or induced pluripotency restart the developmental software program and reset the epigenome

If ageing is the unintended outcome of the running of the developmental software program, both reproduction and cell reprogramming can then be seen as a software restart (Fig. 5). To put it another way, we all originate from a single cell that must reset its software and epigenome to the start of life's program. Indeed, genome-wide chromatin reorganization and epigenetic reprogramming occur postfertilization in the zygote, and during early embryogenesis, to allow toti- or pluripotency [105–107]. Such widespread epigenetic changes during early development—e.g. most methylation is erased and then re-established—have long been seen as a reset [108]. Recent results also show a decrease in epigenetic clocks during early stages of embryogenesis [109]. After restarting, the developmental software program will run with amazing precision during the various stages of development to give rise to many different tissues and cell types. As the program runs, epigenetic changes occur, not randomly or stochastically (although influenced by environmental cues and subjected to biological variation), but largely through a predetermined sequence of events set by the information encoded in the DNA. In later stages of development and in adulthood, as the program runs, so do the epigenetic changes that define the roles and characteristics of a myriad of cells across many different tissues. As the software program runs, the clock ticks in our cells. Even during embryonic development epigenetic age increases [109], again suggesting that the process of ageing is linked to development. Reprogramming an aged cell entails restarting the software, which involves resetting the epigenome (Fig. 5).

Reversal of cellular ageing with Yamanaka factors has even been observed in cells from supercentenarians [110]. Exactly how the software program is reset during reproduction, somatic nuclear transfer and induced pluripotency is not well understood. Even if they are not identical processes, all must involve a reset of epigenetic information and downstream transcriptional regulation, gene expression, and protein changes. In one landmark study, expression of three of the four Yamanaka factors restored youthful epigenetic information and restored vision in aged mice [111]. The authors interpreted these results as evidence that youthful epigenetic information is retained by cells [111], yet an alternative explanation is that, as reprogramming induces a sort of factory reset, the epigenome shifts towards an earlier state in the running of the software and consequently cells are shifted towards a more youthful epigenetic information state.

Not surprisingly, several well-funded companies have been recently set up to harness reprogramming and develop rejuvenation therapies, though many challenges remain [112]. As studies in mice have shown [69, 113], inducing pluripotency with Yamanaka factors in vivo can be harmful, including triggering cancer. The discovery of partial reprogramming [113, 114], that allows cells to be rejuvenated without dedifferentiation, is a promising alternative. Although the mechanisms are poorly understood, partial reprogramming reduces epigenetic age (though not to zero like full reprogramming) and leads to functional improvements in cells [113, 115]. Perhaps partial reprogramming induces a software rewind—and consequently a shift in the epigenome to a previous state in the developmental program—that unlike a software restart (i.e. full reprogramming) maintains cell context. Whether rejuvenated cells, even if differentiated, improve an aged tissue is the, literally, billion-dollar question. The abovementioned study restoring vision in aged mice with reprogramming suggests it is possible [111]. On the other hand, if presbyopia is due to eye lens gradually growing thicker as a run-on developmental process, resetting the developmental program will not reverse the overgrowth. Therefore, restarting or rewinding the developmental software program to rejuvenate cells and achieve clinical benefits in aged tissues holds great promise but will likely require considerable fine-tuning as well as tailoring to specific tissues and degenerative processes.

## Unresolved questions and implications of ageing as a software design flaw

Thus far, I have argued that to understand the ageing process and the majority of what constitutes the ageing phenotype, including various degenerative changes, loss of function in virtually all organs, and predisposition to various diseases, may well lie in studying how residuals of the software program that controls development become detrimental later in life. Progressing from a conceptual grasp of ageing to an in-depth understanding is still a huge and challenging endeavour given how little we understand of the developmental software program that directs how we develop from a single fertilized egg. Indeed, we have only scratched the surface regarding the rules governing and driving ontogeny. So, assuming ageing is a software design flaw, how can we make further inroads into its understanding?

First and foremost, we need to shift from seeing ageing as the outcome of inevitable, spontaneous damage but rather a program. For example, tooth erosion could be seen as a result of wear and tear. On the other hand, as pointed out decades ago by George Williams in his seminal paper introducing antagonistic pleiotropy [48], tooth erosion can be seen a result of their lack of replacement when worn out. In other words, tooth erosion could be interpreted as a design flaw or constraint in the developmental software, even if not one that drives an ageing phenotype but rather fails to prevent it (i.e. an incompleteness in the software); indeed, other species—like some reptiles—feature continuous tooth replacement and thus overcome such design flaw [9]. As such, changing our perspective on ageing has broad implications into designing and interpreting studies and observations.

If ageing phenotypes are embedded in developmental programs, then we need to better integrate developmental data into our models of ageing and age-related diseases. Such an integration is not trivial, even with the plethora of high throughput tools available to us, because of the sheer complexity and volume of developmental changes and because not all developmental processes will impact on ageing. As mentioned before, several omics studies, including on epigenetic clocks [19], have found strong relationships between developmental changes and ageing [10, 49]. We need more detailed studies encompassing the whole lifespan: we need to understand changes in cell composition, changes in particular cell types like stem cells, changes in transcriptional regulation at multiple levels, and eventually unravel how information flows and determines phenotypes across biological space and time. If life is an information system and our life course a collection of transitions—and the rules governing those transitions—between cellular information states then untangling those rules and states is imperative. Ageing is an information problem. To crack ageing, we ultimately need to understand how information encoded in the DNA sequence and memorized in the epigenome instructs a single cell to turn into an embryo, then a foetus that is later triggered by genetic information to become a newborn, it grows to a child, an adult, and then a subset of that information causes it to degenerate, age, and die.

One major implication of the hypothesis proposed here is that cells know how to avoid molecular damage and ageing, but because of design flaws in the developmental software they stop doing so later in life. To elaborate further, some forms of molecular damage to the hardware clearly accumulate with age [8], and increased epigenetic entropy is observed at older ages [45]. Yet, the reason the molecular and cellular hardware gets damaged at older ages is not by and large (cancer being an exception) because of inevitable entropy or overwhelming damage but rather because the way the developmental program runs-on during adulthood. To put it another way, the traditional view is that the hardware accumulates spontaneous, stochastic damage that is not repaired by the software because the repair mechanisms it encodes are inefficient; stochastic damage to the software (i.e. mutations) or data area (i.e. epimutations) can also make them malfunction and cause further damage to the hardware. According to the software design flaw hypothesis, however, there is another option, which is that the instructions in the developmental software later in life lead to damage to the hardware. We thus need to define the changes in information usage in cells that, with age, result in damage and a decline in function.

How exactly, at the cellular and molecular level, the progression of the developmental software program triggers ageing is unclear, however. I speculate it involves a combination of molecular changes, including epigenetic and gene/protein regulation and expression changes that in turn affect cell identity, behaviour, and function (e.g. in stem cells) and lead to high-order changes in tissues and organs, such as changes in metabolism and cell composition, as well as cell-non-autonomous processes that can entail multiple players from signalling molecules to systemic factors like hormones and immune responses. Untangling how and which programs in the developmental software operate and interact at different biological scales from development to adulthood to drive ageing will be a monumental task. Tissue- and organ-specific programs will operate, as well as systemic and cross-tissue interactions like adipose tissues and the immune system. Software design flaws could set trajectories in processes that lead to dysfunction due to either undue increases or decreases later in life of whichever biological factor they regulate [10]; in other words, detrimental processes can include both inappropriate activation of genes or pathways—termed hyperfunction [11, 12]—or inappropriate inactivation—i.e. hypofunction. As mentioned before, a downregulation of repair mechanisms caused by design flaws in the software program cannot also be excluded, which in turn would then lead to the accumulation of molecular damage that might contribute to ageing phenotypes. To be clear, however, such a conceptual model is still very different to traditional damage-based theories of ageing that argue that—because of imperfect maintenance and repair processes—inevitable and stochastic molecular damage causes ageing. By contrast, my hypothesis is that damage (broadly defined in this context) occurs later in life because of changes in cells triggered by the progression of the developmental software program. Put differently, traditional molecular damage hypothesized to drive ageing might be brought about at later ages by the running of the developmental software but will be only one (and, I would speculate, modest) component of the disruption caused by software design flaws. For example, molecular damage is not necessary for presbyopia or thymus involution to occur. Importantly, if we age because of the software’s run-on rather than passive damage to the hardware, then most cellular ageing changes are reversible. Information is suppressed, not lost, during ageing.

Old age is the leading risk factor for pathologies, such as cancer, cardiovascular diseases, type II diabetes, and neurodegenerative diseases, that are the greatest medical challenge of the twenty-first century. Understanding the biology of ageing will shed light on the aetiology of age-related diseases [6]. As such, I suggest that design flaws in the developmental software program contribute to the development of many age-related diseases. Even cancer, which is largely due to molecular damage, is influenced by ageing processes [53, 116]. That is not to say that all age-related diseases are a direct result of the software program or of the ageing process. Most likely there are pathological mechanisms in age-related diseases that are unrelated to other aspects of ageing, as shown at the genetic level [72]. Hence, to understand age-related diseases, it will not suffice to understand ageing biology. That said, individuals are predisposed to age-related diseases because of ageing processes and likely the actions of software design flaws. For example, many infectious diseases, like COVID-19, are much more severe in older patients. One major characteristic of immune system ageing is thymus involution, a process that starts soon after birth and continues throughout life [117], and thus that can be seen as a form of programmatic ageing [13]. Therefore, I speculate that understanding how the developmental software program impacts ageing and predisposes to diseases will have a major impact in our understanding of the aetiology of age-related pathologies.

Seeing ageing as the outcome of software design flaws also has important implications for developing interventions. One prediction from this hypothesis is that traditional anti-ageing interventions targeting damage, like oxidative damage and telomere shortening, will have limited success. They might be beneficial for some specific age-related diseases, but their effects on the ageing process will be limited. By contrast, ageing therapies will only have substantial effects if targeting the software rather than the hardware. Although as abovementioned we know of methods that reset the software, we will likely need interventions safer and more precise than reprogramming—which may well prove too blunt of an instrument—including tissue-specific interventions. Large-scale screening for genes and drugs that modulate epigenetic clocks as well as new cellular rejuvenation methods may pave the way for future interventions. Pharmacological approaches in reprogramming also hold promise [118], but given how embedded ageing is into human biology, I speculate that redesigning life, reprogramming human biology will be necessary to rejuvenate tissues.

## Concluding remarks

If one sees the components of life, such as organs, tissues, cells, proteins, mitochondria, telomeres and even the DNA as hardware, and the instructions in the DNA code as software, contemporary research on ageing has so far assumed that molecular damage to the hardware is the root cause of ageing [5, 7]. Even theories stating that ageing is due to loss of information, like the information theory of ageing [30], posit that damage is the main culprit [31]. This is perhaps intuitive given that inanimate objects accumulate wear and tear that eventually leads to their malfunction. But humans are not inanimate objects. We originate in a single cell that, almost miraculously, divides, grows, and develops to become a fully functional organism. We also continually replace most (though not all) of our individual components. Development is, despite some variability, a well-regulated, deterministic program set by the genome. After reproduction, however, developmental mechanisms have little evolutionary reason to change their predetermined trajectories and hence, I argue, become detrimental. Furthermore, one key facet of the developmental software is epigenetic regulation of gene expression, cell function and cell identity. In a way, the epigenetic state and associated gene regulatory network reflect the running of the developmental software (Fig. 1), the progression of ontogeny, which fits recent findings showing that methylation clocks are highly accurate predictors of biological age from conception until old age [19, 42].

In conclusion, I put forward in this essay the hypothesis that ageing is the outcome of design flaws in the developmental software, events set in motion during development that continue in adulthood and become detrimental later in life (Fig. 2). This hypothesis explains species differences in ageing (Fig. 3) and is in line with the major genetic, dietary, and pharmacological manipulations of ageing (Fig. 4), and it fits recent discoveries in epigenetic clocks and cell reprogramming (Fig. 5). Nonetheless, not all characteristics of the ageing phenotype are caused by unintended consequences of developmental software programs becoming harmful later in life. Cancer, in particular, is mostly driven by genomic damage, and I argue that avoiding cancer is one of the major evolutionary pressures that shaped the developmental software and consequently ageing phenotypes by imposing constraints in adult tissue repair and cell plasticity. Ageing as a software design flaw is a radical departure from damage-based theories that until now have prevailed in biogerontology, and I would argue that the digital ageing code is worthy of further investigation.

## Supplementary Information

Additional file 1. Review history.
