# Supplementary material for: Ageing as a software design flaw
Source: Genome Biol. 2023 Mar 28;24:51. doi: 10.1186/s13059-023-02888-y (PMC10042583; doi:10.1186/s13059-023-02888-y)
Supplement: Supplementary file 1 — Additional file 1. Review history. [file 13059_2023_2888_MOESM1_ESM.docx]

**Review history**

**First round of review**

**Reviewer 1**

I think it is a wonderful and innovative article that will be of interest to anybody who has been puzzled by the question of why we age. The title of the article is a succinct summary of the main hythesis. The author eloquently discusses a provocative hypothesis: intrinsic flaws in the developmental software program are the major driver of what we call ageing. The article is very well written: it is logical and full of illuminating examples that are supported by suitable citations. The author presents a conceptual framework for understanding the many facets of aging and age reversal. This framework integrates many disparate topics: connection between development and aging, comparative biology of lifespan, reprogramming, cancer, and various interventoins that prolong lifespan (dietary interventions, IGF1, rapamycin). I have only very minor edits.
1) Page 7 line 40. Add a sentence to explain the term "developmental plasticity" in the hydra. I assume the "ability to revert development".
2) Page 15 line 15. Reword the following sentence. "For example, many infectious diseases, like COVID-19, are diseases of ageing." Children get COVID as well….
3) If space is a limitation then one could perhaps combine Figure 5 and Figure 1.

**Reviewer 2**

In this review, Prof. Magalhaes puts forward the idea that the ageing process, to some extent, is due to inherent shortcomings of the 'DNA software', as opposed to deterioration or damage of the hardware i.e that is defined as "organs, tissues and the basic unit of life, the cell, and its structures (mitochondria, telomeres, proteins, DNA and so on)". The software could be described as the gene expression programs, and associated epigenetic regulation, that are required for development. However, according to Prof. Magalhaes, these gene expression programs contain inherent 'defects' meaning they cause cellular/molecular deterioration in the context of adult life which manifests as the ageing process.

My background that is relevant to this work is in the field of the epigenetic clock, and not so much in ageing theory, and so I don't know the ageing field in general that well to comment on the novelty of the work, but overall I think this is an interesting article that is generally well written. My comments are below:

1. I am still somewhat confused about the distinction between hardware and software. In particular, when the hardware is damaged e.g. DNA mutations, could the software also not be potentially damaged?

2. There is a very wide body of work on developmental programming effects in utero. The idea being that a sub-optimal in utero environment largely due to dietary or environmental factors (eg smoking) do not alter the DNA sequence but do influence gene expression programs that in turn impact adult onset diseases and phenotypes. I appreciate the rapamycin example, but there is only little discussion about this possibility in the article especially since these effects will indeed influence the software. What is known about how in utero exposures influence the ageing process during adulthood of the directly exposed individual?

3. I disagree with Prof. Magalhaes that "It is indeed remarkable that
neurodegenerative diseases, and other age-related pathologies that are the greatest medical challenge of the 21st century, fails to consider the ageing process as the major driver of those diseases". This is an overstatement and most researchers in the field recognise the importance of ageing in these processes. Furthermore, it is important to remember that diseases such as diabetes are in large part due to changing lifestyles especially in the 20th century. I doubt the software has changed much between now and say 100 years ago, and yet diabetes has reached epidemic proportions and is now not uncommon to see diabetic patients who are still only in their 30s and 40s. In summary, the text that refers to diseases is misleading.

4. As a thought experiment, if we were to consider Conrad Waddington's developmental landscape, could gene expression programs be forced to go down certain routes that would be more beneficial for later life? Therefore, the DNA sequence need not change but the ball could roll down different valleys by certain manipulations e.g. as might occur in developmental programs.

5. The epigenetic clock also supports the idea that different individual cells undergo ageing at different rates, otherwise we would see, for example, certain CpG going from 0 to 100% methylation instantly. This being the case, there has to be some randomness to which genomic regions gain or lose methylation with age and this varies from cell to cell, even though they all ultimately are descendants of the same cell in early development.

5. Finally, might it all be possible that ageing and mortality are not a design flaw at all but rather somehow been selected for at the population level, especially in social animals? I appreciate that there are indeed some organisms that are in principle immortal, but in other contexts, the 'renewal' of the species e very generation allows for selection to act and 'improve' the fitness of the organism? For example, in a closed setting, organisms couldn't just live for ever and keep reproducing as the population would eventually become saturated. Is ageing/death necessarily a 'bad' thing?

6. Finally, I think the text is somewhat unbalanced in the sense that the same question is asked repeatedly - "is it the software"? I appreciate the need to hammer home the point but it might read better by focussing on examples to support the hypothesis as opposed to posing the same question in nearly every paragraph.

**Authors’ response to reviewers**

Reviewer #1: Reviewer #1: I think it is a wonderful and innovative article that will be of interest to anybody who has been puzzled by the question of why we age. The title of the article is a succinct summary of the main hythesis. The author eloquently discusses a provocative hypothesis: intrinsic flaws in the developmental software program are the major driver of what we call ageing. The article is very well written: it is logical and full of illuminating examples that are supported by suitable citations. The author presents a conceptual framework for understanding the many facets of aging and age reversal. This framework integrates many disparate topics: connection between development and aging, comparative biology of lifespan, reprogramming, cancer, and various interventoins that prolong lifespan (dietary interventions, IGF1, rapamycin). I have only very minor edits.

\### I am thankful to the reviewer for the kind comments and useful suggestions, which have helped improve the manuscript.

1) **Page 7 line 40. Add a sentence to explain the term "developmental plasticity" in the hydra. I assume the "ability to revert development".**

\### Thank you for the suggestion, yes, that is indeed the case. I have clarified this in the revised manuscript and cited: Galliot, B., et al. (2006). "Hydra, a niche for cell and developmental plasticity." Semin Cell Dev Biol 17(4): 492‐502.

2) **Page 15 line 15. Reword the following sentence. "For example, many infectious diseases, like COVID‐19, are diseases of ageing." Children get COVID as well….**

\### Thank you, yes, I ended up rewriting the paragraph on diseases based on the comments of reviewer #2 and clarified this point by instead writing that “…infectious diseases, like COVID‐19, are much more severe in older patients.”

3) **If space is a limitation then one could perhaps combine Figure 5 and Figure 1.**

\### Thank you again. I do not think that will be necessary but appreciate the suggestion.

Reviewer #2: In this review, Prof. Magalhaes puts forward the idea that the ageing process, to some extent, is due to inherent shortcomings of the 'DNA software', as opposed to deterioration or damage of the hardware i.e that is defined as "organs, tissues and the basic unit of life, the cell, and its structures (mitochondria, telomeres, proteins, DNA and so on)". The software could be described as the gene expression programs, and associated epigenetic regulation, that are required for development. However, according to Prof. Magalhaes, these gene expression programs contain inherent 'defects' meaning they cause cellular/molecular deterioration in the context of adult life which manifests as the ageing process.

My background that is relevant to this work is in the field of the epigenetic clock, and not so much in ageing theory, and so I don't know the ageing field in general that well to comment on the novelty of the work, but overall I think this is an interesting article that is generally well written. My comments are below:

\### I am thankful to the reviewer for the comments and suggestions, which have helped improve the manuscript.

1. **I am still somewhat confused about the distinction between hardware and software. In particular, when the hardware is damaged e.g. DNA mutations, could the software also not be potentially damaged?**

\### Yes, that is a good point. Indeed, in some theories of ageing, mutations affect the hardware (DNA) and the software is consequently damaged; in the model presented in my paper, however, mutations are not necessary to drive ageing phenotypes – with some exceptions like cancer. In other words, existing models of ageing indeed argue that the software gets corrupted and this leads to malfunction and ageing, while in my model the software’s run‐on leads to malfunction later in life. I have slightly adjusted the definitions of damage, software, and hardware (pages 2 to 4) as well as discussed the above points on mutations and damage to the software in the revised manuscript (pages 15 and 16).

2. **There is a very wide body of work on developmental programming effects in utero. The idea being that a sub‐optimal in utero environment largely due to dietary or environmental factors (eg smoking) do not alter the DNA sequence but do influence gene expression programs that in turn impact adult onset diseases and phenotypes. I appreciate the rapamycin example, but there is only little discussion about this possibility in the article especially since these effects will indeed influence the software.**

What is known about how in utero exposures influence the ageing process during adulthood of the directly exposed individual?

\### Thank you, this is a good suggestion. We do not know very much about gestational influences on ageing, although there is quite a lot of data on its impact on specific age‐related diseases, like type II diabetes. I have mentioned developmental programming in the revised manuscript (page 11) and cited: Langley‐Evans SC (2006). Developmental programming of health and disease. *Proc Nutr Soc*. **65**, 97‐105.

3. **I disagree with Prof. Magalhaes that "It is indeed remarkable that**

neurodegenerative diseases, and other age‐related pathologies that are the greatest medical challenge of the 21st century, fails to consider the ageing process as the major driver of those diseases". This is an overstatement and most researchers in the field recognise the importance of ageing in these processes. Furthermore, it is important to remember that diseases such as diabetes are in large part due to changing lifestyles especially in the 20th century. I doubt the software has changed much between now and say 100 years ago, and yet diabetes has reached epidemic proportions and is now not uncommon to see diabetic patients who are still only in their 30s and 40s. In summary, the text that refers to diseases is misleading.

\### Yes, I agree that was an overstatement. I deleted that sentence and have rephrased and toned down this paragraph on diseases.

4. **As a thought experiment, if we were to consider Conrad Waddington's developmental landscape, could gene expression programs be forced to go down certain routes that would be more beneficial for later life? Therefore, the DNA sequence need not change but the ball could roll down different valleys by certain manipulations e.g. as might occur in developmental programs.**

\### I think this is a very interesting concept, and indeed I would speculate that some manipulations of ageing might be doing exactly that – i.e., forcing gene expression programs down more beneficial routes later in life. It also fits the idea of different developmental programs that are activated or suppressed in different contexts, likely reflecting differences across cells.

5. **The epigenetic clock also supports the idea that different individual cells undergo ageing at different rates, otherwise we would see, for example, certain CpG going from 0 to 100% methylation instantly. This being the case, there has to be some randomness to which genomic regions gain or lose methylation with age and this varies from cell to cell, even though they all ultimately are descendants of the same cell in early development.**

\### Thank you, this is a good point. I agree that there is a certain level of randomness and noise, which seems to increase with age given that variation increases with age at the gene expression and methylation levels. I have briefly mentioned this in the revised manuscript – page 15. There is also a recent review on methylation changes with ageing that discusses these points and that I have cited in the revised manuscript: Seale K, Horvath S, Teschendorff A, Eynon N, Voisin S (2022). Making sense of the ageing methylome. *Nat Rev Genet*. **23**, 585‐605.

5. **Finally, might it all be possible that ageing and mortality are not a design flaw at all but rather somehow been selected for at the population level, especially in social animals? I appreciate that there are indeed some organisms that are in principle immortal, but in other contexts, the 'renewal' of the species e very generation allows for selection to act and 'improve' the fitness of the organism? For example, in a closed setting, organisms couldn't just live for ever and keep reproducing as the population would eventually become saturated. Is ageing/death necessarily a 'bad' thing?**

\### This is an intriguing idea, but I must say that most biologists working on ageing would disagree with it. The most widely accepted evolutionary model is that ageing is detrimental to the individual and hence ageing evolved because of the fading force of natural selection with age. There are exceptions, like the salmon, but in general, ageing is not thought of as an adaptive phenotype. As such, my model draws from the concept of antagonistic pleiotropy proposed by George Williams (Williams GC: Pleiotropy, natural selection, and the evolution of senescence. Evolution 1957, 11:398‐411), and I have tried to make this more explicit on page 6 of the revised manuscript.

6. **Finally, I think the text is somewhat unbalanced in the sense that the same question is asked repeatedly ‐ "is it the software"? I appreciate the need to hammer home the point but it might read better by focussing on examples to support the hypothesis as opposed to posing the same question in nearly every paragraph.**

\### I agree, thank you. I have carefully read the whole manuscript again to remove redundancies and repetition.
